# Firearm exposure and threats among young adults in Texas: Identifying high-risk groups for prevention

**DOI:** 10.1016/j.pmedr.2026.103454

**Published:** 2026-03-21

**Authors:** Jeff R. Temple, Elizabeth R. Baumler, Leila Wood

**Affiliations:** University of Texas Houston Health Science Center, School of Behavioral Health Sciences, Violence and Injury Prevention Research Center, United States

**Keywords:** Firearm access, Firearm victimization, Young adulthood, Educational pathways, Violence prevention, Health disparities, Longitudinal cohort, Non-college youth

## Abstract

**Objective:** Firearm injury is a leading cause of death among adolescents and young adults, yet little is known about firearm access and victimization during this transition. We examine firearm exposure among young adults, as well as disparities across educational pathways, financial strain, and conflict. **Methods:** Data were drawn from Wave 7 (2025) of an ongoing longitudinal cohort study (*n* = 2012; mean age = 20) conducted in southeast Texas. Participants reported firearm access, household gun presence, firearm threat victimization/perpetration, fighting, educational status, and financial stability. Chi-square tests assessed group differences. **Results:** 18% reported firearm access, 24% household gun presence, and 7% firearm threat victimization. Threat victimization was more common among males, non-college and trade-school youth, those experiencing financial hardship, and those involved in physical fighting. **Conclusions:** Young adulthood is a period of elevated firearm vulnerability. Prevention efforts must extend beyond college settings to reach non-college youth and integrate firearm-specific content into existing violence-prevention programs.

## 1 Introduction

Firearm injury is a leading cause of death among adolescents and young adults in the United States (Cunningham et al., 2018; Goldstick et al., 2022; Prevention CfDCa, 2025) Despite this fact, the field lacks developmental data on how young people encounter firearms during the transition into the workforce, trade school, or college. Most firearm research aggregates data across broad age ranges (e.g., 0–17; 10–24; 18–34) (Naik-Mathuria et al., 2023; Fowler et al., 2015) or focuses primarily on children and younger adolescents (Martin et al., 2022; Fowler et al., 2017). Young adulthood (18–24) remains a critical gap in firearm epidemiology (Akande et al., 2025; Goldstick et al., 2024; Taylor et al., 2025). This lack of attention is surprising given that this developmental period is characterized by increased autonomy, mobility, and interpersonal risk behaviors (Arnett, 2000; Arnett et al., 2014; Breiding et al., 2014), as well as higher mortality compared to adolescence (12–17) (Park et al., 2014).

Educational and vocational pathways also represent important but underexamined contexts for firearm exposure. Young adults navigating non-college pathways face elevated risk related to violence, economic precarity, and community instability compared to their college-attending counterparts (Walsemann et al., 2018; Lawrence, 2017; Wood et al., 2023). Yet few studies have quantified firearm-related disparities across individuals who do and do not attend college or trade school. Understanding these patterns is increasingly urgent as young adulthood is marked by widening social and educational stratification in the United States Indeed, young adults associated with Generation Z (1997–2012) are less likely than prior cohorts to achieve financial independence or maintain full time employment (Fry, 2023).

Interpersonal conflict is another key context in which firearm exposure may occur. Youth involved in physical fighting or other conflict-related behaviors often face disproportionately high levels of violence victimization, including firearm-related violence (David-Ferdon et al., 2021). Identifying whether firearm access and gun threat experiences cluster with these behaviors during young adulthood can help clarify which subgroups are most vulnerable and which contexts may require targeted prevention.

To address these gaps, we leveraged a large and ethnically diverse cohort to examine firearm access, household gun presence, and firearm threat victimization and perpetration. By comparing firearm involvement across educational pathways, financial security, and recent interpersonal conflict, we provide developmentally relevant information to guide intervention programs for young adults.

## 2 Methods

### 2.1 Study design and population

Data were drawn from an ongoing longitudinal trial of a healthy relationship intervention (Temple et al., 2021). We recruited participants from middle schools in a large, diverse urban district in 2018 and followed them annually. The cohort was recruited from schools in Southeast Texas, a region characterized by relatively high firearm ownership and permissive firearm policies compared with many other parts of the United States (Lynch et al., 2025). The region includes large urban, suburban, and surrounding rural communities, creating diverse contexts in which young adults may encounter firearms during the transition to adulthood. We limited the current sample to participants who completed Wave 7 (*n* = 2012; Mean age = 20; range = 18–22); fielded February to August of 2025), when firearm questions were introduced. As outlined in Table 1, the sample was diverse.

**Table 1:** Prevalence of firearm access, household gun presence, and firearm threat victimization and perpetration among young adults (ages 18–22) in a longitudinal cohort recruited from middle schools in a large, diverse U.S. metropolitan area, Wave 7 (February–August 2025).

	Sample Profile	Access to a Gun	Gun present in the Home	Been Threatened with a Gun	Threatened Someone with a Gun
	n (%)^1^	n (%)^1^	n (%)^1^	n (%)^1^	n (%)^1^
Overall		356 (18.0)	478(24.3)	146 (7.4)	23 (1.2)
					
Sex					
Male	923 (45.9)	202 (22.3)^⁎⁎⁎^	248 (27.4)^⁎⁎^	95 (10.5)^⁎⁎⁎^	14 (1.5)
Female	1089 (54.1)	154 (14.4)	230 (21.6)	51 (4.8)	9 (0.8)
					
Race					
White	539 (26.8)	108 (20.5)^⁎⁎⁎^	139 (26.4)^⁎⁎⁎^	40 (7.6)^⁎⁎^	5 (0.9)
Black	622 (30.9)	114 (18.8)	162 (26.7)	52 (8.5)	11(1.8)
Asian	407 (20.2)	39 (9.8)	56 (14.0)	13 (3.3)	1 (0.3)
Multiple	110 (5.5)	34 (30.9)	45 (40.9)	14 (12.7)	2 (1.8)
Other	333 (16.6)	61 (18.7)	76 (23.3)	27 (8.2)	4 (1.2)
					
Ethnicity					
Not Hispanic	1193 (59.3)	196 (16.7)	273 (23.3)	75 (6.4)^⁎^	13 (1.1)
Hispanic	818 (40.7)	160 (20.0)	205 (25.7)	71 (8.9)	10 (1.3)
					
Perceived Income					
Live comfortable	886 (44.1)	146 (16.8)	190 (21.9)^⁎⁎^	33 (3.8)^⁎⁎⁎^	9 (1.0)
Meet needs w/ little more	571 (28.4)	114 (20.2)	161 (28.6)	47 (8.3)	6 (1.1)
Just meet basic needs	484 (24.1)	82 (17.3)	117 (24.7)	54 (11.5)	7 (1.5)
Don't meet basic needs	69 (3.4)	14 (21.5)	10 (15.4)	12 (18.5)	1 (1.5)
					
College Enrollment					
Not attending school	683 (34.5)	158 (23.2)^⁎⁎⁎^	174 (25.6)^⁎⁎⁎^	77 (11.3)^⁎⁎⁎^	13 (1.9)
Attending 4-year University	871 (44.1)	115 (13.2)	176 (20.2)	40 (4.6)	4 (0.5)
Attending 2-year College	357 (18.1)	60 (16.9)	107 (30.1)	21 (5.9)	3 (0.8)
Attending Trade School	66 (3.3)	23 (34.8)	21 (32.3)	8 (12.1)	3 (4.5)
					
Physical Fight in the last year					
no	1882 (93.5)	324 (17.2)^⁎⁎⁎^	442 (23.6)^⁎⁎^	118 (6.3)^⁎⁎⁎^	19 (1.0)
yes	83 (4.2)	30 (36.6)	31 (37.8)	27 (32.5)	3 (3.6)

^1^ Valid percentage reported, missing values <2% for all outcomes.

* *p* < 0.05, ** *p* < 0.01, *** *p* < 0.001 from Chi-square test of association across strata, for small cell counts Fisher's exact test was used.

### 2.2 Measures and statistical analysis

We created four yes/no questions to assess firearm access (i.e., “Do you have access to a gun if you needed or wanted one?”), presence in home (i.e., “Do you or does someone living in your home own a gun?”), victimization (i.e., “Have you ever been threatened with a gun?”), and perpetration (i.e., “Have you ever threatened someone with a gun?”). We also assessed past year physical fighting with a yes/no question (i.e., “In the PAST YEAR have you ever been in a physical fight with anyone, not including a dating partner?”), as well as college attendance (i.e., “Are you currently a student at a college or in a post-high school education program?” with the following response options: No; four-year college or university; two-year community or junior college; trade school) and subjective financial status (i.e., “Considering your own income and the income from any people who help you, how would you describe your overall personal financial situation?” with the following response options: live comfortable; meet needs with a little left; just meet basic needs; don't meet basic needs) (Williams et al., 2017). We compared rates using chi-square tests. We obtained active informed consent, and our institutional review board approved this study.

## 3 Results

Overall, 18% of young adults reported having access to a firearm, 24% reported a gun in their household, and 7% reported having been threatened with a gun. As shown in Table 1, males were significantly more likely than females to report access (22% vs 14%,), household gun presence (27% vs 22%), and threat victimization (11% vs 5%). Participants who identified with multiple races were more likely to report access to a firearm (31%), household gun presence (41%), and threat victimization (13%), relative to those who identified as White (21%, 26%, and 8%, respectively), Black (19%, 27%, and 9%, respectively), or Asian (10%, 14%, and 3%, respectively).

Educational pathway differences were evident: 11% of those not attending college and 12% of those attending a trade school had been threatened with a gun, compared to ∼5% of their peers attending two- or four-year colleges. Financial hardship was also associated with increased likelihood of experiencing firearm threats. Finally, firearm threat victimization clustered strongly with physical fighting (33% among those reporting a fight vs. 6% among those who had not).

## 4 Discussion

In this study, we use a large ethnically diverse sample to describe firearm access and gun threat victimization during emerging adulthood – a stage largely overlooked in firearm injury research despite heightened exposure to behavioral, social, and environmental risks (Akande et al., 2025; Goldstick et al., 2024; Taylor et al., 2025; Arnett, 2000; Arnett et al., 2014; Breiding et al., 2014; Park et al., 2014).Approximately one in five young adults in this cohort reported access to a gun, and nearly one in seven males had been threatened with one. Although firearm aggression was rare, firearm victimization was not, underscoring the importance of examining multiple dimensions of firearm involvement to better understand potential health safety impacts. Further, those who identified with multiple races had relatively high rates of access to a firearm and being threatened with one. Although more research is warranted, this latter finding may indicate that multiracial young adults experience unique social and relational contexts (e.g., identity-related stressors, social network patterns) that increase firearm access and threat, independent of patterns observed among monoracial groups.

A key contribution of this study is the identification of educational pathway disparities in exposure to firearms. Youth not enrolled in college or attending trade schools reported substantially higher levels of gun access and gun threat victimization than their peers in two- or four-year colleges. Prior work has documented broader health inequities among non-college young adults (Walsemann et al., 2018; Lawrence, 2017; Wood et al., 2023), but few studies have demonstrated such clear stratification in firearm-related risk. Prevention programs that focus primarily on college campuses risk missing young adults who may be at greatest risk. While these programs must continue, expanding these partnerships with trade schools and workforce training programs may help ensure that violence-prevention efforts reach these populations. Financial hardship was also strongly associated with firearm threat victimization. Economic strain may increase young adults' vulnerability through residing in higher-violence neighborhoods, limited mobility, increased reliance on informal or precarious employment, greater involvement in conflict-prone peer networks, and exposure to chronic stress (Ridley et al., 2020). Future research should examine whether improving stability (e.g., livable wages, cash transfers, affordable housing) reduce firearm threat exposure directly or operate through broader reductions in conflict and environmental risk.

Another important finding is the strong association between involvement in physical fighting and firearm threat victimization. Consistent with established research showing co-occurrence of violence in adolescence (David-Ferdon et al., 2021), young adults who had been in a recent physical fight were more than five times as likely to have been threatened with a gun. This clustering reinforces the idea that firearm encounters may emerge within broader interpersonal conflict dynamics during the transition to adulthood.

These findings have direct implications for adolescent and young adult health promotion. Programs such as the *Fourth R* (Temple et al., 2021), which focus on communication skills, emotion regulation, and conflict de-escalation, are well suited to integrating firearm-specific content. Schools, trade programs, and community agencies could incorporate modules addressing secure firearm storage, navigating peer contexts involving guns, and strategies for nonviolent conflict resolution. Importantly, such programming must extend beyond traditional college settings to reach non-college youth, who appear to be disproportionately exposed to firearm-related risk.

Although this study adds needed developmental data, several limitations should be noted. Firearm measures were self-reported and assessed with single items, which may introduce recall bias, social desirability effects, and ambiguity (e.g., what constitutes “access”). Firearm outcomes were assessed cross-sectionally at Wave 7, limiting our ability to determine temporal ordering or developmental trajectories. The sample, while large and diverse, was drawn from a single metropolitan area in Texas (with relatively loose firearm laws) and may not generalize to rural or regionally distinct firearm contexts, and we did not assess important contextual factors such as storage practices, neighborhood firearm density, or circumstances surrounding threat experiences.

## 5 Conclusion

Despite these limitations, this study highlights the importance of focusing firearm prevention effors on young adulthood. Our findings suggest that firearm access and threat victimization are not evenly distributed across young adults but instead cluster within specific social and educational contexts, particularly among those outside traditional college pathways and those involved in interpersonal conflict. These patterns highlight young adulthood as a critical and underexamined window for firearm prevention. Interventions that extend beyond college campuses and into workforce training programs, trade schools, and community settings may be especially important for reaching young adults who appear most exposed to firearm-related risk.

## CRediT authorship contribution statement

Drs. Temple, Baumler, and Wood contributed to all aspects of the study, including conception and design, acquisition of funding and data, analysis and interpretation, and drafting and revising. All authors approve the final (re)submitted version.

## Funding

All phases of this study were supported by Award Numbers R01HD083445 and 2R01HD083445 (PI: Temple) from the Eunice Kennedy Shriver National Institute of Child Health and Human Development (NICHD) and R01MH129354 (PI: Temple) from the National Institute of Mental Health (NIMH). The content is solely the responsibility of the authors and does not necessarily represent the official views of NICHD or NIMH.

## Declaration of competing interest

The authors declare the following financial interests/personal relationships which may be considered as potential competing interests: Jeff R. Temple, PhD reports financial support was provided by National Institutes of Health. If there are other authors, they declare that they have no known competing financial interests or personal relationships that could have appeared to influence the work reported in this paper.
